# Growth and Etch Rate Study of Low Temperature Anodic Silicon Dioxide Thin Films

**DOI:** 10.1155/2014/106029

**Published:** 2014-02-02

**Authors:** Akarapu Ashok, Prem Pal

**Affiliations:** MEMS and Micro/Nano Systems Laboratory, Department of Physics, Indian Institute of Technology Hyderabad, Medak, Andhra Pradesh 502205, India

## Abstract

Silicon dioxide (SiO_2_) thin films are most commonly used insulating films in the fabrication of silicon-based integrated circuits (ICs) and microelectromechanical systems (MEMS). Several techniques with different processing environments have been investigated to deposit silicon dioxide films at temperatures down to room temperature. Anodic oxidation of silicon is one of the low temperature processes to grow oxide films even below room temperature. In the present work, uniform silicon dioxide thin films are grown at room temperature by using anodic oxidation technique. Oxide films are synthesized in potentiostatic and potentiodynamic regimes at large applied voltages in order to investigate the effect of voltage, mechanical stirring of electrolyte, current density and the water percentage on growth rate, and the different properties of as-grown oxide films. Ellipsometry, FTIR, and SEM are employed to investigate various properties of the oxide films. A 5.25 Å/V growth rate is achieved in potentiostatic mode. In the case of potentiodynamic mode, 160 nm thickness is attained at 300 V. The oxide films developed in both modes are slightly silicon rich, uniform, and less porous. The present study is intended to inspect various properties which are considered for applications in MEMS and Microelectronics.

## 1. Introduction

Silicon dioxide (SiO_2_) thin films are most extensively used insulating films in the manufacturing of silicon-based semiconductor devices, integrated circuits (ICs), and in microelectromechanical systems (MEMS) for different applications such as gate component in metal oxide semiconductor (MOS) transistors, masking layer against diffusion and implantation of dopants in the silicon [1, 2], and isolation of devices [3]. Silicon dioxide thin film is preferred over other dielectric films due to its ease of synthesis, excellent insulating properties, and high quality Si-SiO_2_ interface. Thermal oxidation technique is known to provide high quality SiO_2_ film and hence widely employed [4]. However, high processing temperature (~900–1100°C) of this technique causes redistribution of dopants in silicon substrate during oxide growth and develops stress in silicon substrate that leads to wafer warpage [5–7]. Several new synthesis routes have been developed to reduce the process temperature. Atmospheric pressure chemical vapour deposition (APCVD) [8], plasma enhanced chemical vapour deposition (PECVD) [9], sputtering [10], wet anodic oxidation [11–14], liquid phase deposition (LPD) [15], and sol-gel [16] are few of the low temperature thin film deposition techniques. Each and every process has its own advantages and disadvantages. Among these low temperature techniques, anodic oxidation is one which can be operated even below room temperature [17]. Numerous research groups have investigated anodically grown oxide thin films for gate dielectric component in MOS devices [11, 14, 18]. Anodic oxidation process has several advantages over other techniques such as low cost, simple experimental setup, and room temperature process which minimizes the dopants redistribution and does not involve any toxic and expensive gases. In order to explore anodic oxidation for different applications, further study is required to investigate the properties of as-grown SiO_2_ films with respect to electrolyte solution composition, mode of growth and other relevant processing parameters.

The present work is focused to investigate the effect of applied voltage, electrolyte stirring, current density, and the amount of water on the growth rate, refractive index, and the chemical composition of as-grown oxide thin films. These properties are studied using Ellipsometry, Fourier Transform Infrared Spectroscopy (FTIR), And Scanning Electron Microscope (SEM).

## 2. Experimental Details 

Czochralski (Cz) grown four-inch diameter P-type boron doped (resistivity 1–10 Ω cm) {100} oriented single side polished silicon wafers are diced into 23 × 23 mm^2^ chip size for the deposition of oxide film using anodic oxidation technique. After dicing, chips are cleaned ultrasonically in acetone for 10 minutes followed by thorough rinse in deionized (DI) water. Aluminum is deposited on the rough surface side using e-beam evaporation technique for ohmic contact purpose. Ethylene glycol solvent (purity > 99%, Sigma-Aldrich) mixed with 0.04 M potassium nitrate (KNO_3_) and the certain amount of water is utilized as electrolyte solution. Each time fresh solution is used to avoid the incorporation of glycol by-products in the oxide films. A parallel-plate electrodes system is employed for oxidation process. In the parallel-plate configuration, silicon sample is fixed as anode and the platinum gauge mesh as cathode. A customarily designed sample holder is used to take the contact from the back side of the silicon sample. The design of the chip holder is such that only 2.54 cm^2^ circular area of the sample is exposed to the electrolyte solution. In the experimental setup, anode and cathode are separated by fixed distances of 1.5 cm in potentiodynamic and 2 cm in potentiostatic mode. Prior to oxidation, samples are dipped in 1% hydrofluoric acid (HF) solution until the wetting of the surface is ceased completely to etch out native oxide layer followed by thorough rinsing in DI water to ensure that no traces of HF are left on silicon substrate.

Oxide growth rate is predominantly influenced by water content in the electrolyte, processing parameters such as current density, and temperature of electrolyte. In order to elucidate the effect of these parameters, oxide synthesis is carried out in potentiostatic and potentiodynamic modes with varying water content, current density and voltage. In potentiostatic mode, a fixed quantity (0.5 vol%) of water is added into electrolyte. The oxide synthesis is carried out at a fixed impressed voltage (50–250 V) and continued until the initial current fall down to a minimum current of 2 mA. In the case of potentiodynamic mode, the amount of water is varied from 0 (i.e., no water) to 0.7 vol% and the deposition is performed at a fixed applied current density until the final voltage reaches the predetermined voltage of 300 V. Thereafter, the process is continued in potentiostatic mode at 300 V for 15 minutes. After oxidation, the oxide samples are thoroughly cleaned in DI water to get rid of the adsorbed glycol solvent from the oxide surface. In order to investigate the effect of stirring on oxide film characteristics, the deposition is carried out with and without stirring of electrolyte. In all cases, anodic oxidation is performed at room temperature.

Ellipsometry (J. A. Woolam, model: M-2000D) measurements are performed at three incident angles (65°, 70°, and 75°) to determine thickness and refractive index of the oxide films in the wavelength region of 193–1000 nm. FTIR (Bruker, model: ALPHA) measurement in ATR module is employed to evaluate the chemical bonds present in the oxide films. Scanning electron microscope (ZEISS, model: SUPRA40) is employed to study the surface morphology. The etch rates of oxide films are determined in different types of etchants.

## 3. Results and Discussions

Several characterization techniques are used to study different properties of as-grown oxide films.

### 3.1. Kinetics of Oxide Growth

The kinetics of growth of oxide films is studied for the films deposited in potentiostatic and potentiodynamic modes with varying water content, current density, and voltage.

#### 3.1.1. Potentiostatic Mode

**Figure 1:**
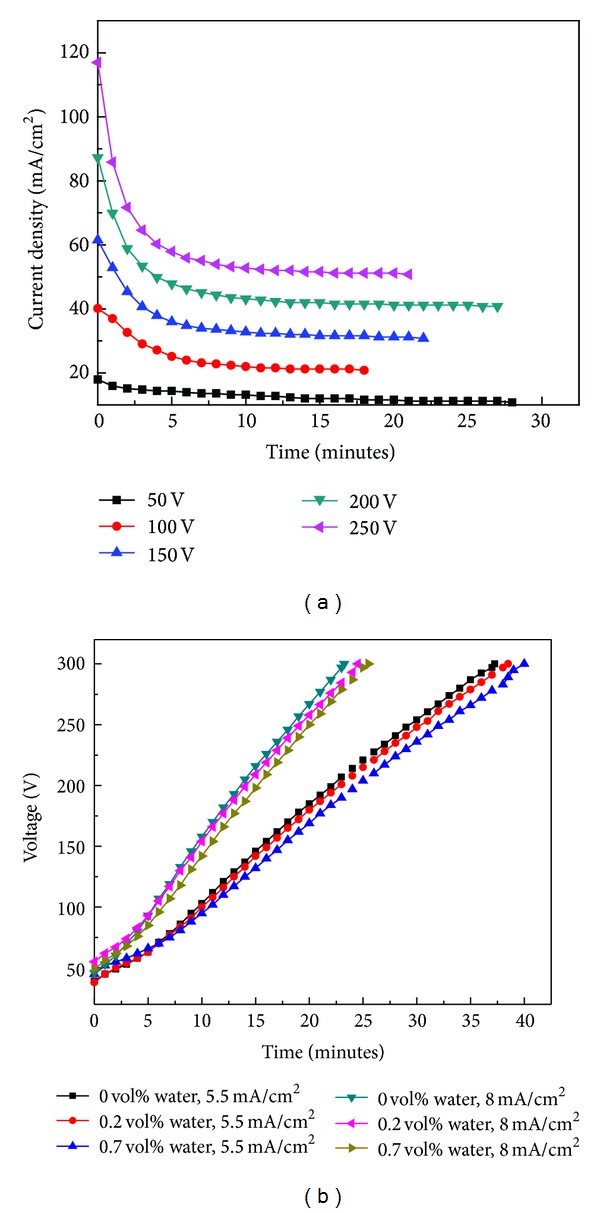
(a) Variation of current density with time in potentiostatic mode at various voltages. (b) Variation of voltage with time in potentiodynamic mode at different current densities and water percentages.

Figure 1(a) shows the variation in current density with oxidation time at various voltages in potentiostatic mode of operation. The shape of the curves suggests that the oxide growth is parabolic at voltages greater than 50 V. It can be observed from Figure 1(a) that the current density at higher voltages decreases rapidly in the first 4 or 5 minutes of oxidation (i.e., initial stage of oxidation) which indicates faster growth rate of oxide in the initial stage of oxidation. After the deposition of certain thickness, the current density becomes almost constant. In this stage, oxide growth rate decreases owing to reduction in the diffusion of oxygenic ions, which happens due to fall in electric field [19, 20].

#### 3.1.2. Potentiodynamic Mode

In potentiodynamic mode, oxide growth is performed at fixed current densities with varying water content in electrolyte. At the fixed current density, voltage increases with time up to the predetermined voltage (300 V) of oxidation process. The variation in cell voltage during oxide deposition is recorded for every 1 min of time and a graph is plotted between anodization time and cell voltage as shown in Figure 1(b). The curves in Figure 1(b) explain that the applied voltage increases in order to maintain the same current density throughout the oxidation process. As can be seen in Figure 1(b), the voltage versus time behaviour is the same for different current densities and water percentages. The role of current density on growth rate of oxide can be understood clearly; for instance, the oxidation time required to attain the predetermined voltage at 8 mA/cm^2^ is shorter than that needed at 5.5 mA/cm^2^. Larger slope indicates faster growth rate at higher current densities which is attributed to enhanced diffusion rate of oxygenic ions [21]. It is obvious from the slope of the curves in Figure 1(b) that the growth rate of the oxide at fixed current density decreases with increase of water percentage in electrolyte, whereas the time required to attain predetermined voltage is reduced with decrease in water percentage. However, the decrease in growth rate is not significant due to the small variation in water content in the electrolyte solution.

### 3.2. Ellipsometric Study

Ellipsometric study is performed to characterize the thickness and refractive index of the films deposited under different conditions.

#### 3.2.1. Thickness

Thickness of oxide films is measured by ellipsometry at three incidence angles (65°, 70°, and 75°). In order to analyze the measured data, a model is generated with two layers, Cauchy type material and an intermediate layer at the interface of Si and SiO_2_.

**Figure 2:**
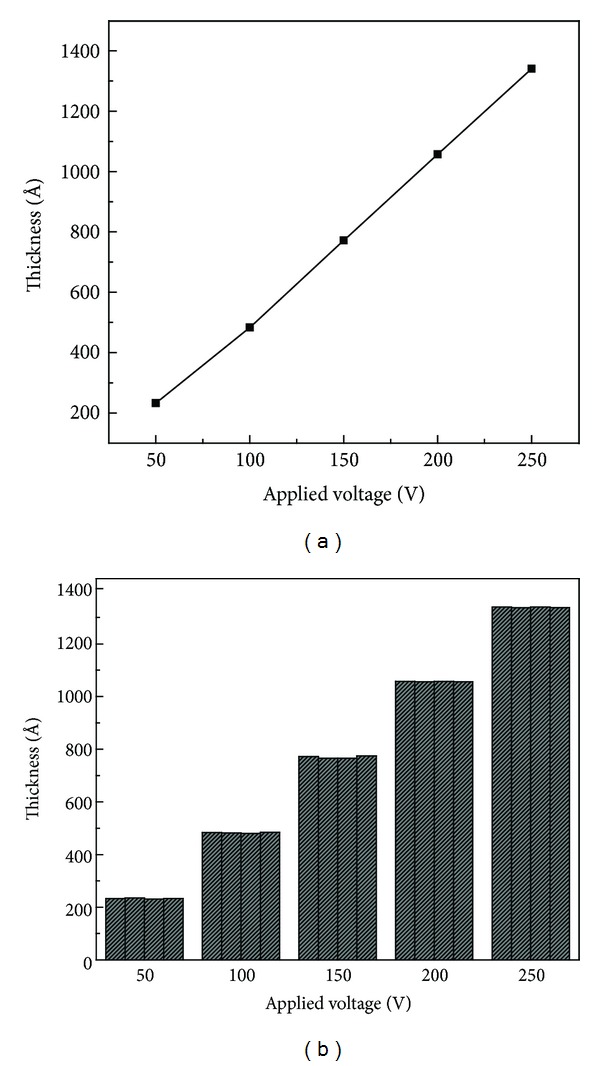
(a) Oxide thickness as a function of voltage in potentiostatic mode. (b) Oxide thickness measured at four different spots to observe thickness uniformity.

Figure 2(a) shows the effect of applied voltage on thickness of the film deposited under potentiostatic mode. It can be noticed from the graph that the thickness increases linearly with applied voltage. Since increase in voltage enhances the driving force of the transport of oxygenic ions through the oxide layer for the growth to happen at Si/SiO_2_ interface [19, 20], the slope of the straight line in Figure 2(a) represents the growth rate of the oxide in Å/V, which is 5.25 Å/V. Similarly, Y intercept gives the native oxide thickness which is zero in this case. Thickness uniformity of the oxide films is evaluated by measuring oxide thickness at four different spots.  Figure 2(b) presents film thicknesses measured at four different spots on the samples prepared under potentiostatic mode at 50–250 V.

**Figure 3:**
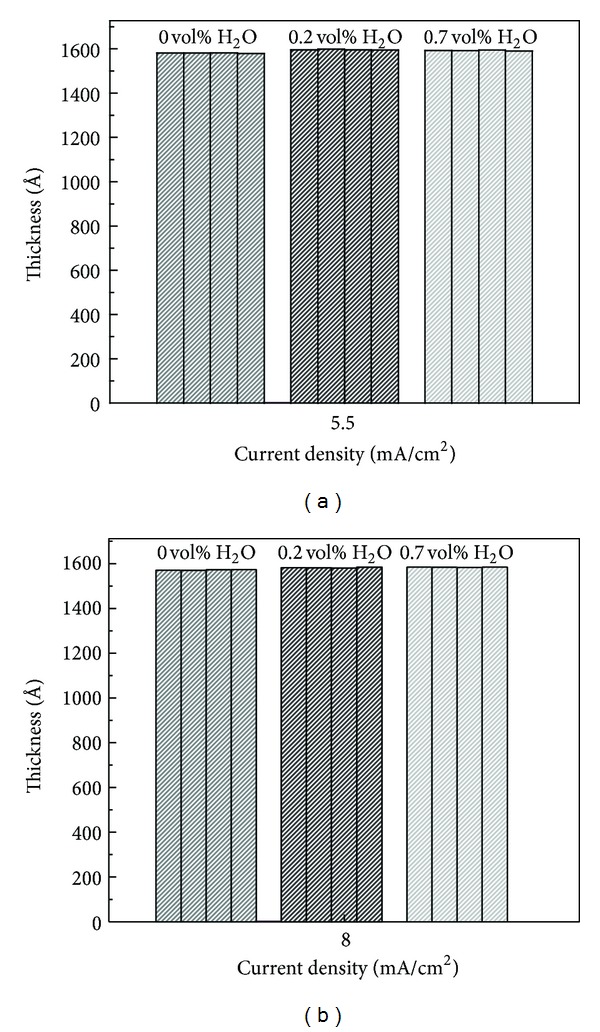
Thickness uniformity check by measuring film thickness at four different spots for the films grown in potentiodynamic mode at (a) 5.5 mA/cm^2^ and (b) 8 mA/cm^2^ with varying water percentages.

Films thicknesses grown under potentiodynamic mode with varying concentration of water in electrolyte at 5.5 and 8 mA/cm^2^ current density are shown in Figures 3(a) and 3(b), respectively. Small thickness variation (1–5 Å) indicates that the films are uniformly grown in potentiostatic and potentiodynamic modes. In potentiodynamic mode, current density does not affect the film thickness significantly as the predetermined voltage (300 V) is the same at 5.5 and 8 mA/cm^2^ current densities. Moreover, the oxide growth is performed on the basis of predetermined voltage not on fixed time period basis.

#### 3.2.2. Refractive Index

Refractive index of oxide films is determined by using variable angle ellipsometry at the fixed wavelength of 632.8 nm. In general refractive index of the oxide is a function of composition or stoichiometry [22–25] and density/porosity of the oxide film [26–28]. The refractive indexes of the oxide films grown in potentiostatic and potentiodynamic modes are presented in Tables 1 and 2, respectively. In potentiostatic mode, the refractive index of the oxides increases from 1.45 to 1.478 as applied voltage varies from 50 to 250 V. The refractive index of the oxide films prepared above 50 V is slightly larger than that of thermally grown silicon oxide. It may be due to more silicon content in the film [24, 29]. Higher silicon content is confirmed by FTIR measurement results, which are presented in the next section, where the small intense peaks of oxygen deficiency that is, Si–Si bond at 653–670 cm^−1^, and suboxides around 988–1000 cm^−1^ are observed [30, 31].

In the potentiodynamic mode of operation, the refractive index of the films deposited with varying water percentage and current density is obtained in the range of 1.474-1.475. Larger refractive index in this mode could be owing to the enhancement in film density (lower porosity) [32, 33] and/or due to oxygen deficiency in the film [24].

### 3.3. Fourier Transform Infrared Spectroscopy (FTIR) Characterization

**Figure 4:**
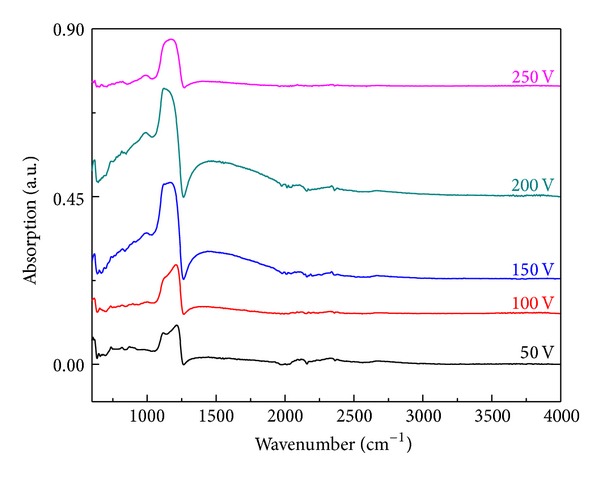
FTIR absorption spectra of oxide films developed under potentiostatic mode at different voltages from 50 to 250 V.

Fourier transform infrared spectroscopy (FTIR) is employed for finding the nature of chemical bonds in the oxide films. In the present work, ATR-FTIR module with 45° angle of light incidence is utilized for the frequency scan of 550–4000 cm^−1^.  Figure 4 shows the comparison of FTIR absorption spectra of the films prepared under potentiostatic mode at various voltages. The characteristic vibrations of SiO_2_ such as Si–O bending and Si–O–Si asymmetric stretching are observed in the wave number range 817–821 cm^−1^ and 1121–1213 cm^−1^, respectively, with Si–O–Si asymmetric stretching peak as intense peak in all samples [30, 34, 35].

The shift in the position of Si–O–Si asymmetric stretching peak occurs due to alteration in oxide thickness, stress/strain, porosity, and the O/Si ratio of the oxide films [24, 27, 36–38]. It can be observed in FTIR spectra shown in Figure 4 that the position of Si–O–Si asymmetric stretching peak is shifting with applied voltage. The applied voltage influences the growth rate, which in turn affects the structure and stoichiometry of the oxides that result in shifting of Si–O–Si asymmetric stretching peak position. In addition to two characteristic Si–O vibrational peaks, weak intense peaks corresponding to oxygen vacancies around 653–678 cm^−1^ (Si–Si bond) [30], carbon impurity (1400–1500 cm^−1^) [39], Si-H (2100–2300 cm^−1^) [40], and silicon suboxide species (SiO_*x*_, *x*~0.5) in the frequency range of 988–1000 cm^−1^ [31] are also present in the spectrum. The absence of the peaks corresponding to Si-OH at 940 cm^−1^ [41] and H-O-H vibrations bending at 1620 cm^−1^ and stretching at 3640 cm^−1^ [41] in the spectra confirms the absence of water in the deposited oxide films.

### 3.4. Effect of Stirring on Various Properties of Silicon Dioxide

**Figure 5:**
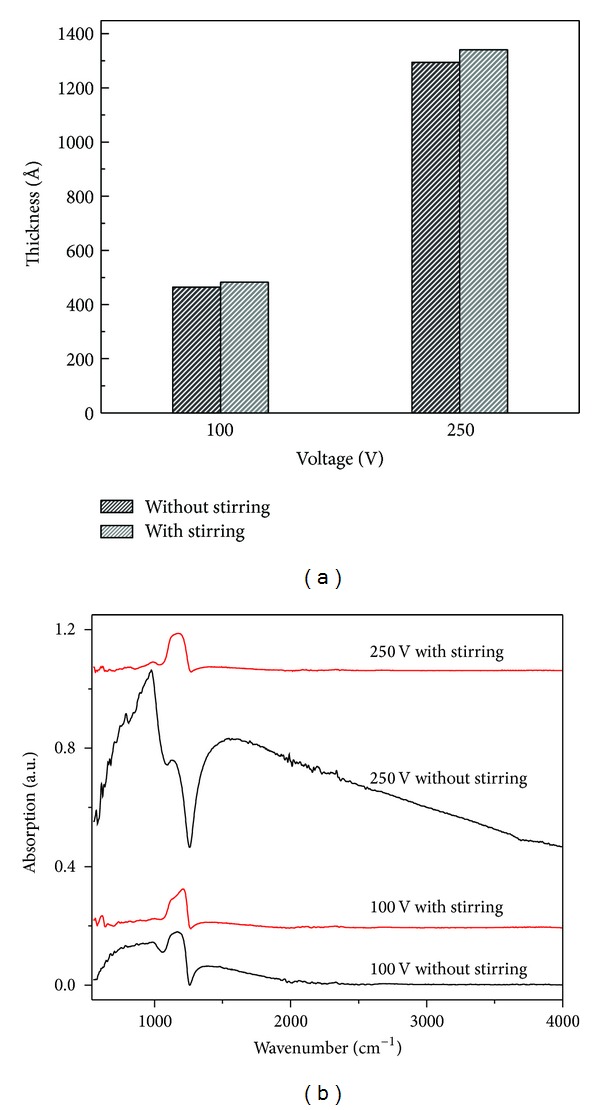
Effect of the mechanical stirring of electrolyte on (a) thickness and (b) chemical structure of oxide films deposited at 100 and 250 V.

Mechanical stirring of electrolyte solution is commonly employed in electrochemical based growth/deposition processes for maintaining uniform concentration of electrolyte throughout the solution and also to circumvent the local temperature rises in the bath. In order to find the effect of electrolyte stirring on thickness and other properties of as-prepared oxide films, depositions are performed with and without mechanical stirring of electrolyte at 100 and 250 V in potentiostatic regime.  Figure 5(a) shows a comparison of film thicknesses grown without and with stirring of electrolyte at two different voltages. The thickness of the film deposited without stirring is about 4% less than that of deposited with stirring of the electrolyte. The reduction in oxide thickness may be due to the lack of convective contribution in the absence of mechanical stirring to the total flux of anionic (OH^−^ or O_2_^−^) species as they are essential for the oxidation to take place at the anode (i.e., silicon) of the cell.

Figure 5(b) shows the comparison of FTIR absorption spectra of oxides grown with and without mechanical stirring at 100 and 250 V. It can be visualized in the spectra that the shape and position of Si–O–Si asymmetric stretching peak differ in two cases of oxidation. This variation may be attributed to the effect of mechanical agitation on the composition and structure of deposited oxides that lead to change in Si–O–Si bond angle [40–42].

The stirring of solution also affects the refractive index as presented in Table 1. The refractive index of the films prepared without stirring of electrolyte at constant voltages 100 and 250 V is slightly less than the films grown with stirring of the solution. This decrease in refractive index could be due to change in composition of the oxides caused by absence of electrolyte stirring.

### 3.5. Surface Morphology Investigation

**Figure 6:**
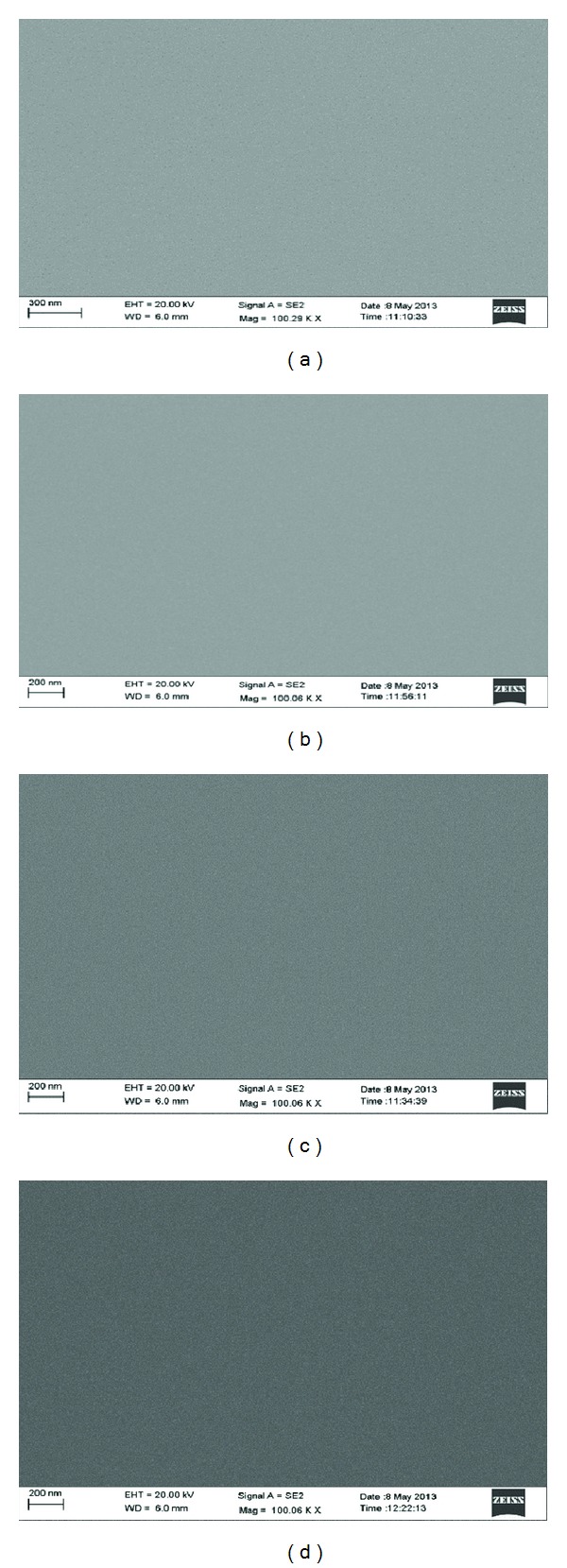
SEM micrographs of the oxide films developed at 0.2 and 0.7 vol% of water (a) and (b) 5.5 mA/cm^2^; ((c) and (d)) 8 mA/cm^2^ current densities in potentiodynamic mode.

Surface morphology of the oxide films is studied using SEM. Figures 6(a)–6(d) reveal the surface morphologies of the oxide films developed in two different electrolyte compositions (0.2 and 0.7 vol% of water) and current densities (5.5 and 8 mA/cm^2^). It can be observed from SEM micrographs that the oxide films do not show any nodular and agglomerated kind of features. The films appear to be smooth and uniform which is also confirmed by ellipsometric measurements discussed in Section 3.2.1. Moreover, no pinholes are noticeable in SEM images that indicate high integrity and density of the oxide films. Similar kind of surface morphologies was observed in the films deposited with different parameters. It can be concluded that the variation in water percentage and current densities employed in this experiment does not significantly influence surface morphology of the films.

### 3.6. Etch Rates in HF-Based Solutions

In order to use silicon dioxide in integrated circuit (IC) and MEMS fabrication, the selective etching of oxide film is required for its patterning. Buffered hydrofluoric acid (BHF) (or diluted HF) is commonly used for oxide etching [42–45]. In the following sections, the study of oxide etching in 1 to 5 wt% HF and the buffered hydrofluoric acid (BHF) solutions is presented. Moreover, the etch rate in diluted HF is also employed for the evaluation of the quality of SiO_2_ film [46].

#### 3.6.1. Diluted HF Solutions

**Figure 7:**
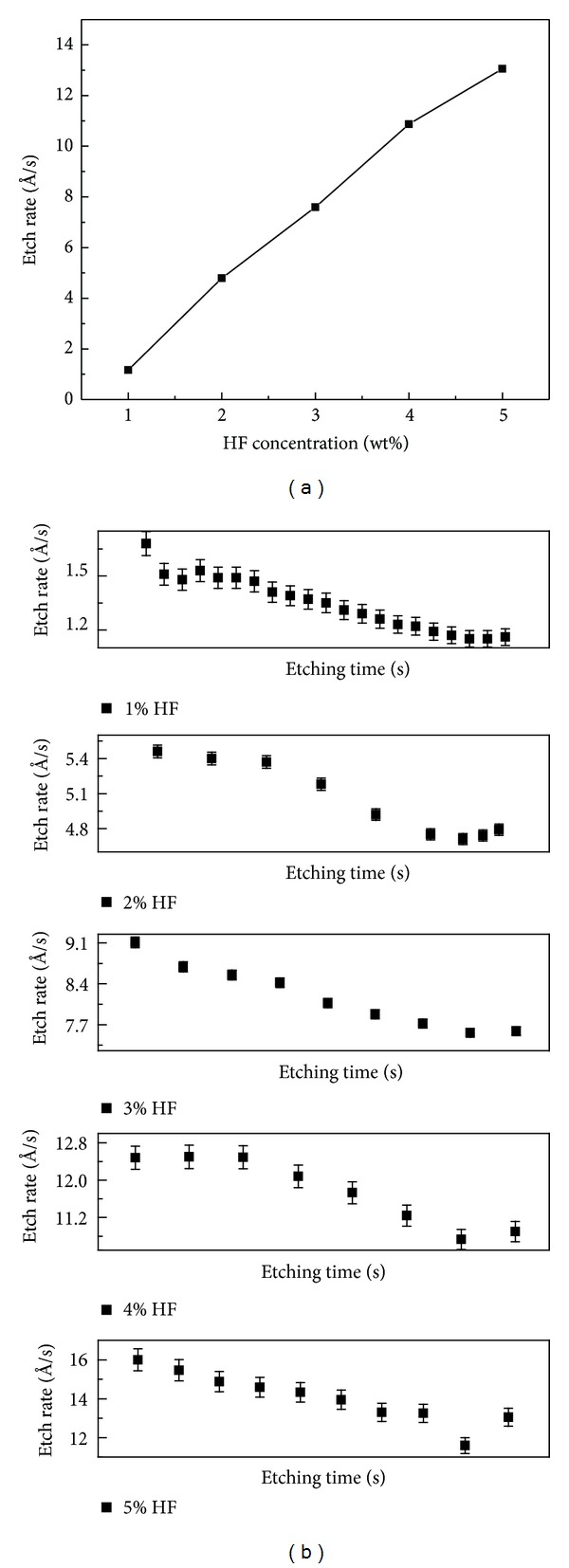
(a) Effect of HF concentration on the etch rate of oxides. (b) Etch rate versus time in different HF concentration for the oxide films grown at 8 mA/cm^2^ current density and 0.7 vol% of water.

Diluted HF is prepared by adding DI water in concentrated HF (i.e., 49 wt%), while BHF is prepared by mixing 40 wt% ammonium fluoride (NH_4_F) in concentrated HF (generally from 6 : 1 to 10 : 1). The etch rate of oxide grown at 8 mA/cm^2^ current density and 0.7 vol% of water (thickness 160 nm) is determined in 1 to 5 wt% HF solutions. Prior to etching, sample is thoroughly cleaned in DI water followed by drying and heating at 100°C for about 5 min. Oxide etching is carried out by immersing the sample in the etchant for fraction of seconds followed by deionized water rinsing. Thereafter, sample is dried and thickness is measured at different spots using ellipsometry. The etching process is continued until oxide thickness is reduced to approximately below 150 Å. The effect of HF concentration on the etch rate is shown in Figure 7(a). The etching mechanism of SiO_2_ in HF-based solutions is explained elsewhere [47]. It can be noticed from the graph that the etch rate of oxide increases almost linearly with HF concentration, which is attributed to enhanced concentration of HF_2_^−^; undissociated HF reactants as the concentration of HF increased up to a maximum at 10 M concentration [48, 49]. The etch rate of oxide is a function of its density, strain, and stoichiometry [47, 50]. It is inversely proportional to the density of the film. The reduced etch rate of oxide in the present work is attributed to greater density (less porosity) of the oxide film, which is confirmed by SEM study as shown in Figure 6(d).

Figure 7(b) shows the etch rate behavior of oxide with time for HF concentrations varying from 1 to 5 wt%. It can be noticed from the figure that the trend in etch rate variation with time is the same for all HF concentrations. The variation in etch rate is probably due to change in oxide structure, that is, Si–O–Si bond angle [51].

#### 3.6.2. Etch Rate in Buffered Hydrofluoric Acid (BHF) Solution

Etch rate of the oxide film (thickness 160 nm) grown at 8 mA/cm^2^ and 0.7 vol% water is measured in BHF (HF (49%) : NH_4_F (40%):: 1 : 7) [45]. The addition of NH_4_F to HF increases the concentration of HF_2_^−^ reactants in the etchant. The etch rate of oxide increases with NH_4_F concentration up to the maximum at 10–12% of NH_4_F [52, 53]. The procedure of etch rate determination in BHF is the same as explained in the previous section for diluted HF. Average etch rate in BHF for the oxide film developed in the present work is measured to be 93.0 Å/sec.

## 4. Conclusions 

Silicon dioxide thin films with uniform thickness are developed on silicon samples using anodic oxidation process at room temperature. In potentiostatic mode of anodic oxidation, thickness predominantly depends on applied voltage. A linear dependency of oxide thickness on applied voltage is obtained in the range from 50 to 250 V. The mechanical agitation of electrolyte solution during deposition improves the growth rate and the chemical structure of oxide films. In potentiodynamic mode, oxide growth rate is mainly influenced by current density irrespective of the amount of water in the electrolyte. The growth rate increases with rise in current density. No significant effect is observed on growth rate, thickness, refractive index and the surface morphology of the films when the water content in electrolyte is varied by a small amount. The FTIR spectra of the oxides developed in potentiostatic mode reveals that the oxide films contain small percentage of carbon, hydroxyl impurities and the absence of OH and H_2_O. The dense nature of the oxide films is confirmed by measuring the etch rate in diluted HF (1 to 5 wt%) and using SEM micrographs.

## Figures and Tables

**Table 1:** Refractive index of the oxide films prepared in potentiostatic mode with and without mechanical stirring of the electrolyte solution.

Regime: potentiostatic, pH = 3, water content = 0.5 vol%	
Applied voltage (*V*)	Refractive index (*n*) (with/without stirring)
50	1.45
100	1.470/1.466
150	1.473
200	1.475
250	1.478/1.474

**Table 2:** Refractive index of the oxide films developed in potentiodynamic mode.

Regime: potentiodynamic, pH = 4		
Current density (mA/cm^2^)	Water content (vol%)	Refractive index (*n*)
5.5	0	1.4755
8	0	1.4756
5.5	0.2	1.4748
8	0.2	1.4749
5.5	0.7	1.4747
8	0.7	1.4752

## References

[1] Robles S, Yieh E, Nguye BC (1995). Moisture resistance of plasma enhanced chemical vapor deposited oxides used for ultralarge scale integrated device applications. *Journal of the Electrochemical Society*.

[2] Pierson HO (1992). *Handbook of Chemical Vapor Deposition (CVD): Principles, Technology and Applications*.

[3] Zhang C, Najafi K (2004). Fabrication of thick silicon dioxide layers for thermal isolation. *Journal of Micromechanics and Microengineering*.

[4] Wu W-F, Chiou B-S (1996). Optical and mechanical properties of reactively sputtered silicon dioxide films. *Semiconductor Science and Technology*.

[5] Grove AS, Leistiko O, Sah CT (1964). Redistribution of acceptor and donor impurities during thermal oxidation of silicon. *Journal of Applied Physics*.

[6] Deal BE, Grove AS, Snow EH, Sah CT (1965). Observation of impurity redistribution during thermal oxidation of silicon using the MOS structure. *Journal of the Electrochemical Society*.

[7] Yorium Y (1982). Deformation of silicon wafers by thermal oxidation. *Journal of the Electrochemical Society*.

[8] Pecora A, Maiolo L, Fortunato G, Caligiore C (2006). A comparative analysis of silicon dioxide films deposited by ECR-PECVD, TEOS-PECVD and Vapox-APCVD. *Journal of Non-Crystalline Solids*.

[9] Thompson MJ (1984). Thin film transistors for large area electronics. *Journal of Vacuum Science & Technology B*.

[10] Bhatt V, Chandra S (2007). Silicon dioxide films by RF sputtering for microelectronic and MEMS applications. *Journal of Micromechanics and Microengineering*.

[11] Hung TF, Wong H, Cheng YC, Pun CK (1991). New design of anodic oxidation reactor for high-quality gate oxide preparation. *Journal of the Electrochemical Society*.

[12] Lewerenz HJ (1992). Anodic oxides on silicon. *Electrochimica Acta*.

[13] Duffek EF, Mylroie C, Benjamini EA (1964). Electrode reactions and mechanism of silicon anodization in *N*-methylacetamide. *Journal of the Electrochemical Society*.

[14] Águas H, Gonçalves A, Pereira L, Silva R, Fortunato E, Martins R (2003). Spectroscopic ellipsometry study of amorphous silicon anodically oxidised. *Thin Solid Films*.

[15] Kim KS, Roh Y (2007). Silicon dioxide deposited by using liquid phase deposition at room temperature for nanometer-scaled isolation technology. *Journal of the Korean Physical Society*.

[16] Kambhampati DK, Jakob TAM, Robertson JW, Cai M, Pemberton JE, Knoll W (2001). Novel silicon dioxide sol-gel films for potential sensor applications: a surface plasmon resonance study. *Langmuir*.

[17] Panagopoulos C, Badekas H (1989). Growth of anodic SiO_2_ films. *Materials Letters*.

[18] Kim W-B, Matsumoto T, Kobayashi H (2009). Ultrathin SiO_2_ layer with an extremely low leakage current density formed in high concentration nitric acid. *Journal of Applied Physics*.

[19] Sun L, Zhang S, Sun XW, He X (2009). Effect of electric field strength on the length of anodized titania nanotube arrays. *Journal of Electroanalytical Chemistry*.

[20] Mei Y-F, Wu X-L, Qiu T, Shao X-F, Siu G-G, Chu PK (2005). Anodizing process of Al films on Si substrates for forming alumina templates with short-distance ordered 25 nm nanopores. *Thin Solid Films*.

[21] Soliman HMA, Kashyout A-HB (2011). Electrochemical deposition and optimization of thermoelectric nanostructured bismuth telluride thick films. *Engineering*.

[22] Ayupov BM, Devyatova SF, Erkov VG, Semenova LA (2008). Depth profiles of refractive index in thermally grown and LPCVD oxide films on silicon. *Russian Microelectronics*.

[23] Ceiler MF, Kohl PA, Bidstrup SA (1995). Plasma-enhanced chemical vapor deposition of silicon dioxide deposited at low temperatures. *Journal of the Electrochemical Society*.

[24] Lin C-F, Tseng W-T, Feng MS (2000). Formation and characteristics of silicon nanocrystals in plasma-enhanced chemical-vapor-deposited silicon-rich oxide. *Journal of Applied Physics*.

[25] Roschuk T, Wojcik J, Tan X, Davies JA, Mascher P (2004). Optical and compositional characterization of SiO_x_N_y_ and SiO_x_ thin films deposited by electron cyclotron resonance plasma enhanced chemical vapor deposition. *Journal of Vacuum Science & Technology A*.

[26] Stadtmueller M (1992). Mechanical stress of CVD-dielectrics. *Journal of the Electrochemical Society*.

[27] Lucovsky G, Manitini MJ, Srivastava JK, Irene EA (1987). Low-temperature growth of silicon dioxide films: a study of chemical bonding by ellipsometry and infrared spectroscopy. *Journal of Vacuum Science & Technology B*.

[28] Joshi BN, Mahajan AM (2007). Growth and characterization of porous SiO_2_ thin films for interlayer dielectrics applications in ULSI. *Optoelectronics and Advanced Materials*.

[29] Pliskin WA (1987). Refractive index dispersion of dielectric films used in the semiconductor industry. *Journal of the Electrochemical Society*.

[30] Alayo MI, Pereyra I, Scopel WL, Fantini MCA (2002). On the nitrogen and oxygen incorporation in plasma-enhanced chemical vapor deposition (PECVD) SiO_x_N_y_ films. *Thin Solid Films*.

[31] Lambers J, Hess P (2003). Infrared spectra of photochemically grown suboxides at the Si/SiO_2_ interface. *Journal of Applied Physics*.

[32] Sassella A, Lucarno P, Borghesi A, Corni F, Rojas S, Zanotti L (1995). Silicon oxynitride study by the tetrahedron model and by spectroscopic ellipsometry. *Journal of Non-Crystalline Solids*.

[33] del Pradoa A, San Andrés E, Martínez FL (2002). Composition and optical properties of silicon oxynitride films deposited by electron cyclotron resonance. *Vacuum*.

[34] Galeener FL (1979). Band limits and the vibrational spectra of tetrahedral glasses. *Physical Review B*.

[35] Pai PG, Chao SS, Takagi Y, Lucovsky G (1986). Infrared spectroscopic study of SiO_x_ films produced by plasma enhanced chemical vapor deposition. *Journal of Vacuum Science & Technology A*.

[36] Pliskin WA, Lehman HS (1965). Structural evaluation of silicon oxide films. *Journal of the Electrochemical Society*.

[37] Pliskin WA (1977). Comparison of properties of dielectric films deposited by various methods. *Journal of Vacuum Science & Technology*.

[38] Boyd IW, Wilson JIB (1987). Structure of ultrathin silicon dioxide films. *Applied Physics Letters*.

[39] Tedder LL, Crowell JE, Logan MA (1991). The chemical vapor deposition of SiO_2_ from tetraethoxysilane: the effect of the surface hydroxyl concentration. *Journal of Vacuum Science & Technology A*.

[40] Tsu DV, Lucovsky G, Davidson BN (1989). Effects of the nearest neighbors and the alloy matrix on SiH stretching vibrations in the amorphous SiO_r_:H (0 < *r* < 2) alloy system. *Physical Review B*.

[41] Liao W-S, Lin C-H, Lee S-C (1994). Oxidation of silicon nitride prepared by plasma-enhanced chemical vapor deposition at low temperature. *Applied Physics Letters*.

[42] Monk DJ, Soane DS, Howe RT Sacrificial layer SiO_2_ wet etching for micromachining applications.

[43] Monk DJ, Soane DS, Howe RT (1993). Determination of the etching kinetics for the hydrofluoric acid/silicon dioxide system. *Journal of the Electrochemical Society*.

[44] Judge JS (1971). A study of the dissolution of SiO_2_ in acidic fluoride solutions. *Journal of the Electrochemical Society*.

[45] Kikuyama H, Waki M, Kawanabe I (1992). Etching rate and mechanism of doped oxide in buffered hydrogen fluoride solution. *Journal of the Electrochemical Society*.

[46] Kern W, Schnable GL, Moss SJ, Ledwith A (1987). Wet etching. *The Chemistry of the Semiconductor Industry*.

[47] Monk DJ, Soane DS, Howe RT (1993). A review of the chemical reaction mechanism and kinetics for hydrofluoric acid etching of silicon dioxide for surface micromachining applications. *Thin Solid Films*.

[48] Deckert CA (1978). Etching of CVD Si_3_N_4_ in acidic fluoride media. *Journal of the Electrochemical Society*.

[49] Parisi GI, Haszko SE, Rozgonyi GA (1977). Tapered windows in SiO_2_: the effect of NH_4_F: HF dilution and etching temperature. *Journal of the Electrochemical Society*.

[50] Schwettmann FN, Dexter RJ, Cole DF (1973). Etch rate characterization of boron-implanted thermally grown SiO_2_. *Journal of the Electrochemical Society*.

[51] Choi YW, Ahn JH, Ahn BT (2005). Structure of SiO_2_ films grown at low temperature by inductively coupled plasma oxidation with oxygen gas. *Electronic Materials Letters*.

[52] Buhler J, Steiner FP, Baltes H (1997). Silicon dioxide sacrificial layer etching in surface micromachining. *Journal of Micromechanics and Microengineering*.

[53] Proksche H, Nagorsen G, Ross D (1992). Influence of NH_4_F on the etch rates of undoped SiO_2_ in buffered oxide etch. *Journal of the Electrochemical Society*.

